# Subtle neuropsychiatric and neurocognitive changes in hereditary gelsolin amyloidosis (AGel amyloidosis)

**DOI:** 10.7717/peerj.493

**Published:** 2014-07-22

**Authors:** Mari Kantanen, Sari Kiuru-Enari, Oili Salonen, Markku Kaipainen, Laura Hokkanen

**Affiliations:** 1 Helsinki University Central Hospital (HUCH), Department of Psychiatry, Hospital District of Helsinki and Uusimaa, Finland; 2 Helsinki University Central Hospital (HUCH), Department of Neurology, Hospital District of Helsinki and Uusimaa, Finland; 3 Department of Clinical Neurosciences, University of Helsinki, Finland; 4 Helsinki University Central Hospital (HUCH), Department of Radiology, Hospital District of Helsinki and Uusimaa, Finland; 5 Institute of Behavioural Sciences, Division of Cognitive Psychology and Neuropsychology, University of Helsinki, Finland

**Keywords:** Hereditary amyloidosis, Gelsolin amyloidosis, Neuropsychological testing, Visuoconstructional problems, Depression, Magnetic resonance imaging

## Abstract

Hereditary gelsolin amyloidosis (AGel amyloidosis) is an autosomal dominant form of systemic amyloidosis caused by a c.640G>A or c.640G>T mutation in the gene coding for gelsolin. Principal clinical manifestations include corneal lattice dystrophy, cranial neuropathy and cutis laxa with vascular fragility. Signs of minor CNS involvement have also been observed, possibly related to cerebral amyloid angiopathy (CAA). To investigate further if AGel amyloidosis carries a risk for a specific neuropsychological or psychiatric symptomatology we studied 35 AGel patients and 29 control subjects. Neuropsychological tests showed abnormalities in visuocontructional and -spatial performance in AGel patients, also some indication of problems in processing efficacy was found. At psychiatric evaluation the patient group showed more psychiatric symptomatology, mainly depression. In brain MRI, available in 16 patients and 14 controls, we found microhemorrhages or microcalcifications only in the patient group, although the number of findings was small. Our study shows that AGel amyloidosis can be associated with visuoconstructional problems and depression, but severe neuropsychiatric involvement is not characteristic. The gelsolin mutation may even induce cerebrovascular fragility, but further epidemiological and histopathological as well as longitudinal follow-up studies are needed to clarify gelsolin-related vascular pathology and its clinical consequences.

## Introduction

Hereditary gelsolin amyloidosis (AGel amyloidosis) is an autosomal dominant form of systemic amyloidosis, caused by a c.640G>A or c.640G>T (earlier known as G654A or G654T) gelsolin gene mutation. The disorder was originally identified in Finland in the late 1960s (Meretoja, 1969), but has since been reported from several countries in Europe, the United States, Japan, Iran and Brazil (Kiuru-Enari & Haltia, 2013). The age of disease onset is in the third or fourth decade of life. The three dominating clinical findings are corneal lattice dystrophy, cutis laxa, and cranial neuropathy, but signs of CNS involvement have also been observed, possibly related to gelsolin-related cerebral amyloid angiopathy (CAA) (Kiuru et al., 1995; Kiuru, Salonen & Haltia, 1999).

CAA is characterized by progressive deposition of amyloid in cortical, subcortical and leptomeningeal vessels, which can cause leucoencephalopathy, intracerebral hemorrhages (ICH), cerebral microbleeds (CMB), ischemic infarcts, and dementia or cognitive dysfunction (Charidimou, Gang & Werring, 2012; Chao, Kotsenas & Broderick, 2006; Koennecke, 2006; Yamada, 2000). The diagnosis of CAA is neuropathologic, but the diagnosis can be reached by brain imaging methods MRI and CT-data showing CAA-related pathology particularly in the cortical-subcortical areas and in the white matter (Charidimou, Gang & Werring, 2012; Chao, Kotsenas & Broderick, 2006; Koennecke, 2006). Several sporadic and hereditary cerebrovascular amyloid proteins and types of CAA have been identified (Charidimou, Gang & Werring, 2012; Yamada, 2000; Revesz et al., 2009). Genetic risk factors for developing sporadic CAA are the apolipoprotein E (ApoE) alleles, which have an important role in amyloid-beta metabolism, and are reported to influence an increased risk for the CAA and CAA related ICH (Charidimou, Gang & Werring, 2012; Biffi et al., 2010). Rare hereditary forms of CAA are based on mutations in the gene encoding the amyloid precursor protein (APP), and the clinical manifestations differ from the sporadic type of CAA (Charidimou, Gang & Werring, 2012; Haan et al., 1990). In gelsolin-related CAA the amyloid distribution pattern has been distinct, predominant in smaller blood vessels (Kiuru, Salonen & Haltia, 1999). The prevalence of sporadic CAA is reported to be higher in the elderly population and it is strongly associated with Alzheimer’s disease (AD) (Arvanitakis et al., 2011; Keage et al., 2009; Pfeifer et al., 2002; Farlow et al., 1992), but it is also observed in elderly subjects without AD pathology (Arvanitakis et al., 2011; Soffer, 2006).

Through its association with vascular and white matter changes, it has been suggested that CAA is an independent risk factor for clinically important cognitive dysfunction (Greenberg et al., 2004). A study by Arvanitakis et al. (2011) (*n* = 404) showed that after controlling for age, sex, education, AD-pathology, infarct and Lewy bodies, moderate-to very severe CAA was associated with lower performance in perceptual speed and episodic memory. Multiple cortical infarcts also had higher odds of dementia. No associations of mild-to-moderate CAA with cognition were found. Cerebral microbleeds (CMB) have been associated with deficits of executive functions independent of subtype of ischemic stroke, white-matter lesions, hypertension and age (Werring et al., 2004). Vascular and white matter pathology are known to be associated with impaired recall, processing speed and executive functions, but also neuropsychiatric symptoms particularly depression specifically in the elderly has been reported (Hommet et al., 2011). It has been suggested that vascular pathology and white matter lesions may be associated with pathogenesis of depression and depressive symptoms by disrupting the neural circuits between the frontal and subcortical regions (Naarding et al., 2005; Thomas et al., 2002). A previous study of AGel amyloidosis with CNS involvement reported widespread spinal, cerebral and meningeal amyloid angiopathy with deposition of AGel. A study by Kiuru, Salonen & Haltia (1999) in four patients with a G654A gelsolin mutation revealed widespread areas of diffuse signal increase in cerebral white matter, particularly in the frontal lobes and the pons, in addition cerebral and a few cerebellar white-matter hypertensities were seen. Histological findings revealed alterations of the white matter and involvement of spinal and cerebral blood vessels and meninges, and slight to moderate diffuse loss of myelin (Kiuru, Salonen & Haltia, 1999). Dementia is reported in single cases (Kiuru, Salonen & Haltia, 1999; Darras et al., 1986; Haltia et al., 1991), potentially related to associated CNS diseases. In the study of 31 AGel patients by Kiuru et al. (1995) minor signs of CNS involvement were found in the MRI revealing significantly more frequent high intensity lesions in subcortical and deep white matter, periventricular regions and/or in the pons. Also, abnormal evoked potentials compared to the controls were reported. In the same study the neuropsychological assessment in 30 AGel patients revealed subtle impairment in abstract thinking, cognitive flexibility and visuoconstructional and -spatial difficulties, suggesting CNS abnormalities, possibly with predominant frontal involvement. Depressive symptoms were also reported.

The rationale behind searching neurocognitive changes in AGel amyloidosis stems from two sources. Firstly, the CAA pathology is strongly associated with Alzheimer’s disease (Arvanitakis et al., 2011; Keage et al., 2009; Pfeifer et al., 2002; Farlow et al., 1992), and because of its association with vascular and white matter changes, CAA may be an independent risk factor for cognitive dysfunction also in non-AD patients (Greenberg et al., 2004). Therefore, the possibility of mild cognitive decline or dementia should be investigated. Secondly, earlier studies have tentatively suggested signs of CNS involvement especially in the fronto-subcortical regions in familial amyloidosis (Kiuru et al., 1995). The prefrontal lobes give rise to three neuronal circuits affecting cognitive functions, behaviour and mood; the dorsolateral prefrontal circuit mediates the organization of information to facilitate a response; the anterior cingulate circuit is required for motivated behaviour, and the orbitofrontal circuit is involved in the integration of limbic and emotional information into behavioural responses (Cummings, 1990; Bonelli & Cummings, 2007). Impaired executive functions, apathy, and impulsivity are hallmarks of frontal-subcortical circuit dysfunction and therefore both neuropsychological and neuropsychiatric approach is warranted.

The aim of the present study is to investigate CNS abnormalities in AGel amyloidosis. A group of AGel patients is assessed using neuropsychological, neuropsychiatric and neuroradiological methods and compared with healthy controls. Our hypothesis is that there are subtle fronto-subcortical manifestations that affect cognitive and emotional performance.

## Methods

### Participants

A total number of sixty-four (64) subjects, 35 AGel patients and 29 age-matched healthy controls participated in the study. Four of the AGel patients were participants of an earlier study by the same authors (Kiuru et al., 1995; Kiuru, Salonen & Haltia, 1999), others were novel cases. There were no differences in age, education and gender between two subject groups (Table 1).

**Table 1:** The demographic information of the AGel patient and the control group. The description of number of cases, mean and standard deviation (SD) of age and education years.

	AGel patients	Controls
	*n* = 35	*n* = 29
Female/male	23/12	18/11
Age	58.09 (10.41)	57.41 (11.07)
Range	31–74	32–77
Education	12.60 (2.47)	13.03 (2.73)

The participants were recruited from the Department of Neurology in Helsinki University Central Hospital with patients’ age-matched healthy relatives with no amyloidosis symptoms serving as a control group. Written informed consent was obtained from all participants. This study was approved by the Ethics Committee for the Obstetrics and Gynaecology, Paediatrics and Psychiatry, Hospital District of Helsinki and Uusimaa, Finland, approval number HUS 213/E7/05.

The diagnosis of AGel amyloidosis was based on clinical examination, typical clinical findings or demonstration of the G654A gelsolin gene mutation in patients (Paunio et al., 1992). Detailed medical histories (neurologic and/or psychiatric diseases, diseases predisposing to cerebrovascular disorders), use of CNS-affecting medication and alcohol were obtained. The exclusion criteria were age under 18 years, pregnancy or other diagnosed CNS-diseases.

### Psychiatric assessment

All participants filled out the Beck Depression Inventory-II (BDI-II) (Beck, Steer & Brown, 1987; Beck, Steer & Brown, 2004), and the Symptom Check List-90 (SCL-90) (Derogatis, Lipman & Covi, 1973; Holi, Sammallahti & Aalberg, 1998; Holi, Marttunen & Aalberg, 2003). The structured diagnostic psychiatric interview (Structured Clinical Interview for DSM Disorders, SCID I and SCID II) (First et al., 1996; First et al., 1997) was used to evaluate the current psychiatric diagnoses and psychiatric episodes in the past five years of the patient and control participants. The structured interview was carried out without blinding of the diagnostic group by psychiatrist Markku Kaipainen.

### Neuropsychological assessment

A battery of selected neuropsychological tests was used to cover a wide range of basic and higher-level cognitive functions. All assessments were carried out without blinding of the diagnostic group by the first author MK in one session.

Verbal and non-verbal abstract thinking and strategic reasoning were measured by four Wechsler Adult Intelligence test-Revised (WAIS-R) (Wechsler, 1981; Wechsler, 1992) subtests Information, Similarities, Picture Completion and Block design. In the analyses we used the raw scores, age serving as a covariate. The WAIS-R Verbal Intelligence Quotient (VIQ) was estimated using the Information and the Similarities subtests. The Picture Completion and the Block design were used to estimate the WAIS-R Performance Intelligence Quotient (PIQ).

To assess memory functions and learning we used the Logical Memory and the Visual Reproduction subtests of the Wechsler Memory Scale-Revised (WMS-R) (Wechsler, 1987; Wechsler, 1996), and the Word list and the Letter-number Sequencing subtests of the Wechsler Memory Scale-III (Wechsler, 1997; Wechsler, 2007). In the analysis we used the raw scores, age serving as a covariate.

Attention, cognitive flexibility and processing efficacy we assessed with Trail Making Test (TMT) parts A & B, the Stroop test (Stroop, 1935; Jensen & Rohwer, 1966), the Paced Auditory Serial Addition Test (PASAT) (Gronwall, 1977), and the Digit symbol subtest of the WAIS-R. In TMT the total test completion time and the number of errors were measured in parts A and B separately (Lezak, Howieson & Loring, 2004). In addition, to evaluate influence of speed in the test performance the difference score TMTb-TMTa was calculated (Corrigan & Hinkeldey, 1987). In the Stroop test (100 items) we used the parts Colour Naming (Part II) and Colour–Word Interference (Part III). In both parts, the total test completion time, and the amount of errors and errors corrected were scored (Lezak, Howieson & Loring, 2004). In addition, the total test completion time difference between Parts II and III (Part II–Part II) was calculated (Lezak, Howieson & Loring, 2004; Corrigan & Hinkeldey, 1987).

Visuoconstructional and visuospatial abilities were evaluated by paper- and pencil-drawing tests (Lezak, Howieson & Loring, 2004; Christensen, 1974; Caplan & Romans, 1998) and the CERAD Clock Drawing task (Consortium to Establish a Registry for Alzheimer’s Disease) (Pulliainen et al., 1999; Sotaniemi et al., 2012). The test performance in the visuoconstructional drawing task was scored from 1 to 3 (no abnormality, slight and severe abnormality). In the CERAD Clock Drawing the overall shape, location of numbers, clock hands and their direction are each scored yielding a total score ranking from 1 to 6.

### Neuroradiological methods

Due to limited resources brain MRI was carried out to the first 16 patients (12 female and 4 men, mean age 55.25 (SD = 9.89, range 38–74)) and the 14 first control subjects (10 female and 4 men, mean age 57.07 (SD = 10.81, range 41–77)) recruited in the study. Brain MRI was performed on 1.5 T machine (Siemens, Erlangen, Germany). All persons had T2-weighted (axial and sagittal plane), fast-FLAIR and T2* (axial plane), T1-weighted sagittal and DW-images. All brain abnormalities including infarcts and bleedings were recorded without blinding of the diagnostic group by the author OS. The WMHIs were classified as periventricular WMHIs or deep WMHIs and graded 0 through 3. The severity of white matter lesions (WMLs) was rated with the Fazekas’ scale (Fazekas et al., 1987).

### Statistical analysis

A multivariate analysis of covariance (MANCOVA) with age as a covariate was used for the statistical analysis of differences in the psychiatric data of patient and control groups using SPSS version 21 (IBM Corp., 2012). The subsequent univariate analyses were carried out using the independent samples *t*-test or the analysis of covariance (ANCOVA) adjusted for multiple comparisons using the Bootstrap method (R multtest package (R Core Team, 2013; Pollard et al., 2013)). The neuropsychological tests were analysed in three separate MANCOVA blocks: one for logical reasoning/intelligence measures, one for memory measures and one for processing speed and accuracy measures. In these multivariate analyses age was used as a covariate, and the subsequent univariate analyses were carried out using the Bootstrap method. Power analysis for the differences of means of the psychiatric and neuropsychological measures was carried out based on the two samples’ *t*-tests (R pwr package). This was done without adjusting for age. Additionally, a logistic regression analysis was conducted to examine the prediction of group membership (AGel patients or controls) with the neuropsychological measured. To avoid multicollinearity, only 13 neuropsychological variables with low inter-correlations were chosen for the analysis. All predictor variables, along with age, were simultaneously entered into the model. Chi-square-test was used for statistical analysis of discrete data, such as the differences in the visuoconstructional performance. Unless otherwise stated, all statistical comparisons are two-tailed. Descriptive statistics are given as means and standard deviations (SD).

## Results

### Neuropsychiatric findings

Table 2 shows the psychiatric symptomatology measured by SCL-90 and BDI-II self-report inventories in the two groups. In an overall MANCOVA with all the subscales as well as the BDI-II included and age used as a covariate, there was no significant effect of group (Wilks’ Lambda = .755, *F*(12, 50) = 1.35, partial *η*^2^ = .245) and the scores were relatively low in both groups. Univariate comparisons revealed however that the somatic symptom subscale of the SCL-90 was slightly more elevated in the patient group compared to the control group (*F*(1, 61) = 8.07, adjusted *p* = .03).

**Table 2:** The current psychiatric symptoms reported by the patient and the control group. The current symptom means and standard deviations (SD) using the SCL-90 and BDI-II self-report inventories. Also, statistical differences between the groups as well as the power of the analyses are presented.

	AGel patients	Controls	*p*	Adjusted *p*	Partial *η*^2^	Power
	Mean (SD)	Mean (SD)				
SCL-90 GSI^*^	1.66 (.47)	1.49 (.45)	0.17	0.56	.031	0.29
Somatic	2.09 (.65)	1.65 (.56)	0.006	0.03	.117	0.78
ocd	2.04 (.81)	1.88 (.78)	0.43	0.95	.010	0.12
Interpersonal	1.56 (.55)	1.51 (.57)	0.74	1.00	.002	0.06
Depression	1.83 (.73)	1.59 (.64)	0.19	0.60	.029	0.26
Anxiety	1.50 (.47)	1.37 (.44)	0.27	0.77	.020	0.19
Aggression	1.32 (.40)	1.33 (.39)	0.93	1.00	.000	0.05
Fobic	1.71 (.26)	1.14 (.28)	0.66	1.00	.003	1.00
Paranoia	1.34 (.42)	1.36 (.47)	0.86	1.00	.001	0.05
Psychotic	1.26 (.28)	1.22 (.29)	0.65	1.00	.003	0.08
Additional	1.99 (.68)	1.74 (.62)	0.13	0.46	.037	0.31
BDI-II total	7.46 (8.16)	3.79 (5.95)	0.048	0.21	.062	0.48

**Notes.**

* SCL-90 Global Severity Index.

The results of the diagnostic psychiatric interview showed more lifetime clinical psychiatric disorders in the patient group. From the total of 35 patients, 10 had a clinically relevant psychiatric disorder: nine had depression and one an anxiety disorder. In one case the depression was diagnosed within one year before the AGel amyloidosis, in all other cases the depression was in the temporal sense secondary to the AGel amyloidosis diagnosis. In the control group (*n* = 29), one case of clinical depression and one of anxiety disorder was found. The frequency of depression within the AGel patient group (26%) compared with the control group (3%) was significantly higher (chi-square = 5.96, *p* = 0.015).

### Neuropsychological findings

Table 3 shows the logical reasoning and the memory measures in the patient and the control groups. Results were at an average or above average level on both groups. In MANCOVA, with age used as a covariate, no significant effect of group was found in the verbal (VIQ) or the performance intelligence (PIQ) or in the subtests of the WAIS-R (Wilks’ Lambda = .828, *F*(6, 56) = 1, 94, partial *η*^2^ = .172). However, in subsequent analyses there was a difference between the groups in the WAIS-R block design-subtest, the patient group performing worse than the control group (*F*(1, 61) = 7, 12; adjusted *p* = .032).

**Table 3:** Logical reasoning and memory performance of the AGel patient and the control group. The mean and standard deviation (SD) of the Wechsler Adult Intelligence Scale (WAIS-R) verbal and performance IQ, as well as the raw scores of the WAIS-R and Wechsler Memory Scale (WMS) subtests. Also, statistical differences between the groups as well as the power of the analyses are presented.

	AGel patients	Controls	*p*	Adjusted *p*	Partial *η*^2^	Power
	Mean (SD)	Mean (SD)				
WAIS-R verbal IQ	115.11 (13.50)	112.90 (12.39)	0.52	0.94	.007	0.10
WAIS-R performance IQ	117.94 (12.71)	122.55 (14.02)	0.21	0.59	.025	0.26
WAIS-R information	25.49 (3.94)	24.59 (4.22)	0.39	0.85	.012	0.13
WAIS-R similarities	27.40 (3.48)	26.66 (3.62)	0.42	0.87	.011	0.13
WAIS-R picture completion	17.34 (1.96)	17.31 (2.71)	0.87	1.00	.000	0.05
WAIS-R block design	29.77 (9.06)	35.10 (8.24)	0.01	0.03	.104	0.64
WMS-R stories immediate recall	28.03 (5.74)	26.02 (7.05)	0.34	0.90	.015	0.23
WMS-R stories delayed recall	25.06 (6.27)	24.07 (7.75)	0.50	0.98	.007	0.08
WMS-R figures immediate recall	36.43 (4.46)	37.00 (3.95)	0.64	1.00	.004	0.08
WMS-R figures delayed recall	29.86 (9.98)	29.69 (11.45)	0.86	1.00	.000	0.05
WMS-III wordlist A total score	32.17 (5.79)	31.86 (6.77)	0.74	1.00	.002	0.05
WMS-III wordlist A interference	8.03 (2.12)	7.38 (2.91)	0.25	0.80	.021	0.17
WMS-III wordlist A delayed recall	7.17 (2.43)	7.00 (3.19)	0.71	1.00	.002	0.06
WMS-III letter-number sequencing	10.34 (2.95)	10.93 (2.87)	0.45	0.96	.009	0.12

The performance in verbal and visual memory and learning were at an average level in both groups. In the MANCOVA with all the memory subscales, and age used as a covariate, no significant effect of group was found (Wilks’ Lambda = .919, *F*(8, 54) = .594, partial *η*^2^ = .081) and the subsequent univariate analyses revealed no further differences. There were no statistical differences in immediate or delayed memory or learning between the patient and control groups.

No significant difference in the processing speed between the groups was found. In the MANCOVA with all the tests of executive functions and attention, and age used as a covariate, there was no significant effect of group (Wilks’ Lambda = .809, *F*(8, 54) = 1, 59, partial *η*^2^ = .191). In univariate comparisons there was an indication of faster performance of the AGel group in the Stroop Colour–Word Interference test and in the Stroop III–II time difference, but these differences were not significant after adjusting for multiple comparisons (see Table 4). Additionally, there were some indications of differences between the groups in processing efficacy and accuracy. Univariate comparisons revealed that the sum of errors in all these tests was slightly elevated in the patient group compared to the control group, but the difference was not statistically significant.

**Table 4:** Processing speed and accuracy in the AGel patient and the control groups. The means and standard deviations (SD) in the tests of processing speed and accuracy in the two groups. Also, statistical differences between the groups as well as the power of the analyses are presented.

	AGel patients	Controls	*p*	Adjusted *p*	partial *η*^2^	Power
	Mean (SD)	Mean (SD)				
TMA^*^	48.17 (19.16)	48.17 (15.57)	0.88	1.00	.000	0.05
TMB^*^	112.69 (48.45)	110.55 (50.79)	0.99	1.00	.000	0.05
TMB–A^*^	64.51 (35.65)	62.38 (45.80)	0.93	1.00	.000	0.06
Stroop II (colour naming)^*^	80.43 (15.38)	85.10 (18.43)	0.23	0.83	.024	0.19
Stroop III (colour-word interference)^*^	122.34 (21.09)	139.97 (34.13)	0.01	0.09	.102	0.68
Stroop III–II	41.91 (14.51)	54.86 (25.63)	0.01	0.11	.096	0.69
PASAT correct	41.83 (13.51)	42.97 (10.15)	0.76	1.00	.001	0.07
PASAT errors	5.34 (3.72)	3.76 (2.69)	0.06	0.29	.057	0.44
WAIS-R digit symbol	42.49 (13.01)	44.93 (10.59)	0.58	0.96	.005	0.12
Errors (TM and Stroop) total	6.77 (4.53)	4.72 (3.28)	0.04	0.21	.067	0.49

**Notes.**

* Seconds.

The logistic regression analysis with 13 neuropsychological variables revealed a significant model with 83% of all cases correctly classified (chi-square = 32.2, *p* = 0.004). Two predictors were significant: WAIS-R Block design and total sum of errors in TM and Stroop (see Table 5).

**Table 5:** Logistic regression analysis for the prediction of group membership (AGel patients or controls). Odds ratios (OR) as well as 95% confidence intervals (CI) for the OR are given.

	*B*	S.E.	OR	95% CI		Wald	*p*
				Lower	Upper		
Constant	−0.75	7.00				0.01	0.91
Age	0.07	0.05	1.07	0.96	1.19	1.51	0.22
WAIS-R information	−0.17	0.13	0.84	0.65	1.10	1.61	0.21
WAIS-R similarities	0.05	0.14	1.05	0.79	1.38	0.10	0.75
WAIS-R picture completion	−0.23	0.21	0.80	0.53	1.19	1.23	0.27
WAIS-R block design	0.27	0.08	1.31	1.12	1.54	11.03	0.001
WMS-R stories immediate recall	−0.05	0.08	0.95	0.81	1.11	0.41	0.52
WMS-III letter-number sequencing	0.25	0.18	1.29	0.91	1.82	2.02	0.15
WMS-R figures immediate recall	−0.17	0.14	0.85	0.64	1.12	1.39	0.24
WMS-R figures delayed recall	−0.09	0.05	0.92	0.84	1.01	3.14	0.08
WMS-III wordlist A total score	0.01	0.09	1.01	0.85	1.21	0.02	0.90
TMB^*^	0.01	0.01	1.01	0.99	1.04	0.68	0.41
PASAT correct	0.00	0.04	1.00	0.92	1.09	0.00	0.99
Stroop III (colour-word interference)^*^	0.02	0.02	1.02	0.99	1.05	1.87	0.17
Errors (TM and Stroop) total	−0.34	0.14	0.71	0.55	0.93	6.24	0.01

**Notes.**

* Seconds.

There were differences in visuoconstructional abilities in the drawing tasks, showing more errors or difficulties in the patient group (chi-square = 7.96, *p* = .014) using exact test (Table 6, see also Supplemental Information S1). In the clock-drawing task there was no significant difference in the performance of the patient and the control group; the mean score of the patient group was 5.37 (SD 0.91) and the controls 5.52 (SD 0.63).

**Table 6:** Visuoconstructional copying in the AGel patients and the control group. The qualitative scoring and the differences in the visuoconstructional copying task between the AGel patients and the control group. See also Supplemental Information S1.

	AGel patients (*n* = 35)	Controls (*n* = 29)
No abnormality (*n*)	11	18
Slight abnormality (*n*)	22	8
Severe abnormality (*n*)	2	3

### Neuroradiological findings

Four of the 16 patients had either single microhemorrhage or microcalcification on the MRI while none of the 14 control subjects had them (chi-square = 4.04, one-tailed *p* < .022). One patient had multiple hemorrhages, and one patient had a sign of a small old infarct. No significant differences in WMHI or atrophy ratings were found between patients and age-matched control subjects. The neuropsychological test results and the psychiatric status of the AGel patients with microhemorrhages are shown in Table 7. There was no clear association between microhemorrhages/microcalcifications and specific neuropsychological or neuropsychiatric symptoms on the group level. However, when the raw scores are compared with the scores of the control group (Tables 3 and 4) it can be seen that patient A had some difficulty in delayed verbal memory (WMS-R stories and WMS-III wordlist) as well as in processing accuracy and speed (TM and Stroop), patient C in processing accuracy (PASAT and errors total) and patient D in visual delayed memory (WMS-R figures) and attention (WMS-III letter-number sequencing, WAIS digit symbol and errors total). Patients C and D also had clinically relevant psychiatric disorder, depression and anxiety, and all patients (A to D) reported more depression related symptoms compared with controls (see Table 2). Patient A had been diagnosed with hypertension two years prior to the AGel amyloidosis diagnosis. Patients B, C and D had no cerebrovascular predisposing factors in their medical histories.

**Table 7:** The neuropsychological test results and the psychiatric status of the four AGel patients with microhemorrhages in the MRI.

	Patient A	Patient B	Patient C	Patient D
Sex (female/male)	F	F	F	M
Age (years)	60	68	59	59
BDI-II	10	11	9	9
Psychiatric diagnosis	No	No	Depression	Anxiety
WAIS-R verbal IQ	96	133	120	99
WAIS-R performance IQ	120	116	127	130
WMS-R stories immediate recall	25	26	26	30
WMS-R stories delayed recall	19	25	22	25
WMS-R figures immediate recall	34	38	39	38
WMS-R figures delayed recall	31	38	32	21
WMS-III wordlist A total score	29	27	33	28
WMS-III wordlist A interference	5	9	10	6
WMS-III wordlist A delayed recall	4	6	8	7
WMS-III letter-number sequencing	7	10	11	2
Visuoconstructional drawings (1–3)	2	2	2	2
Clock drawing (0–6)	5	5	5	6
TMA	48	64	78	42
TMB	175	126	142	98
TMB–A	127	62	64	56
Stroop part II (colour naming)	92	94	76	85
Stroop part III (colour-word interference)	159	110	119	137
Stroop Part III–II	67	16	43	52
PASAT correct	31	52	38	–
PASAT errors	3	8	11	–
WAIS-R digit symbol	34	40	35	29
Errors total	6	8	11	12

## Discussion

In the present study we examined the possible CNS involvement in AGel amyloidosis using a controlled study design. Our aim was to pay special attention to findings associated with the disruption of the fronto-subcortical circuits. The present data show subtle indication of neuropsychiatric, neurocognitive and MRI findings associated with AGel amyloidosis supporting our hypothesis. The patient group reported more depression and somatic symptoms than the control group and showed more clinically relevant psychiatric disorders, mainly depression. In neuropsychological testing, we found some indication of problems in processing efficacy and accuracy, and more clearly in visuoconstructional or visuospatial abnormality in AGel group. On the MRI, the findings were elevated in the patient group, showing either microhemorrhages or microcalcifications.

There were minor differences in the current psychiatric symptomatology between the groups in self-report measures, patients reporting more somatic symptoms than the controls. There was no clear difference in the current self-reported symptoms of depression. In clinical psychiatric evaluation assessing both past and present situation however the patient group showed clinically relevant psychiatric disorders, mainly depression, more frequently. The depression-related symptomatology may partly be reactive to the AGel disease and its effects to functional ability and quality of life. The somatic symptoms reported by the patients, such as reduced sleep or lack of energy, may be associated with the clinical manifestations of the AGel amyloidosis or they can reflect the somatic component of depression. Since vascular and white matter pathology are associated with pathogenesis of depression and depressive symptoms (Naarding et al., 2005), our findings raise the question of possible organic pathology behind the depressive symptomatology found mainly in the patient group.

In the neuropsychological assessment no differences between the patient and the control group were found in the verbal or the performance intelligence or memory functions. Even in the individual cases with neuroradiological findings, the cognitive profile remained well preserved, and there was no sign of rapid forgetting (immediate vs. delayed memory scores compared), one of the hallmarks of Alzheimers’ disease. It can be concluded that in this form of AGel amyloidosis, dementia does not appear to be prevalent. Similar was the finding also in the previous study of 31 cases (Kiuru et al., 1995), although dementia has been reported in single patients (Kiuru, Salonen & Haltia, 1999; Darras et al., 1986; Haltia et al., 1991).

Subtle differences in the neuropsychological performance were discovered however. The visuoconstructional difficulties of the patient group were evident both in the block design test and in the drawing task. The overall processing speed was equal in both groups, but the patient group showed a tendency towards more errors in their responses. The patients were currently no more depressed than the controls so that did not explain the findings. In fact, the control group was slower in the Stroop test, and it appears that the AGel patients traded accuracy for speed and failed to control for errors. The processing and response accuracy may reflect problems in action control and executive functions. Also, in addition to visual processing, the constructional performance is strongly associated with executive functions such as planning, organization of action and cognitive flexibility, all requiring frontal involvement. Difficulties in processing accuracy, action control and visuoconstructional performance may be an indicator of organic pathological changes in frontal or fronto-subcortical circuits. In previous studies on CAA, cerebral microbleeds and cognition, lower perceptual or processing speed, problems in executive functions, poor episodic memory and impaired recall have been the main neuropsychological findings reported (Arvanitakis et al., 2011; Greenberg et al., 2004; Poels et al., 2012; Gregoire et al., 2012).

Microhemorrhages are considered to be associated with cerebral small vessel pathologies and CAA. In our study the MRI showed differences between the groups as microhemorrhages and microcalcifications were mainly found in the AGel patient group. There were no significant differences in WMHI or atrophy ratings between patients and control subjects. The group was also too small for any clear associations to emerge between microhemorrhages or microcalcifications and specific neuropsychological or psychiatric symptoms. The four individual AGel patients with MRI-findings showed mild cognitive problems in processing and memory functions as well as psychiatric symptomatology but this finding should be viewed with caution due to the qualitative nature of the case analysis.

It has been suggested that CAA and its clinical manifestations are more common in the elderly population. In our study we examined participants with a mean age under 60 and it is possible that the changes we found on the MRI and in the neurocognitive functions could be more evident in more elderly study subjects.

Our study has two notable limitations. The sample size is relatively small and therefore the statistical power in the analyses remained low. Possibly with larger samples more differences would have emerged. AGel amyloidosis however is an uncommon disease and collection of large study groups in a low population country is very time consuming. We believe that by reporting these findings we can enhance more international research in the area. The second limitation is the un-blinded method of data collection. It can be hypothesized that the knowledge of the group membership can affect the overall impression of the rater resulting in exaggeration of the negative findings in patients. As AGel amyloidosis is a disease often resulting in noticeable changes in the skin tissue (cutis laxa), particularly in the facial area, blind evaluation is difficult to accomplish. Relying on global impressions or interview data alone is therefore unadvisable. Objective measures should always be included, as was done in our study.

In conclusion, previous studies have suggested minor CNS abnormalities in AGel amyloidosis, possibly related to cerebral amyloid angiopathy (CAA). Our findings conducted in a controlled study design confirm this and indicate subtle changes in the MRI, neurocognitive function and psychiatric symptomatology in the AGel group compared to age-matched control subjects. Longitudinal studies are needed to investigate whether these findings are progressing in the course of the illness.

## Supplemental Information

10.7717/peerj.493/supp-1 Supplemental Information S1 File S1 Visuoconstructional drawings of the four patients described in Table 6, (a, b, c and d, pages 1 to 4, respectively) with slight abnormality, as well as the 2 patients (pages 5 and 6) and 3 controls (pages 7 to 9) with severe abnormality. Subjects were asked to copy the Greek cross and the cube, and to fill in the missing lines of the cubes at the bottom of the page.
