# Supplementary material for: Subtle neuropsychiatric and neurocognitive changes in hereditary gelsolin amyloidosis (AGel amyloidosis)
Source: PeerJ. 2014 Jul 22;2:e493. doi: 10.7717/peerj.493 (PMC4121541; doi:10.7717/peerj.493)

A

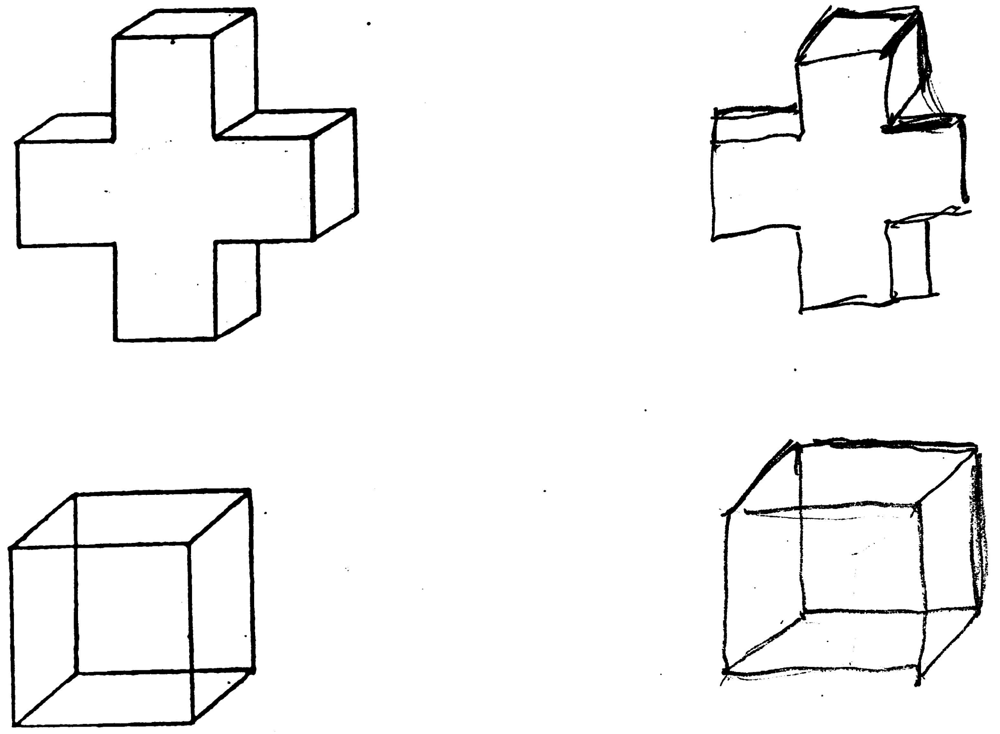

Täydennä alla olevat kuutiomallit:

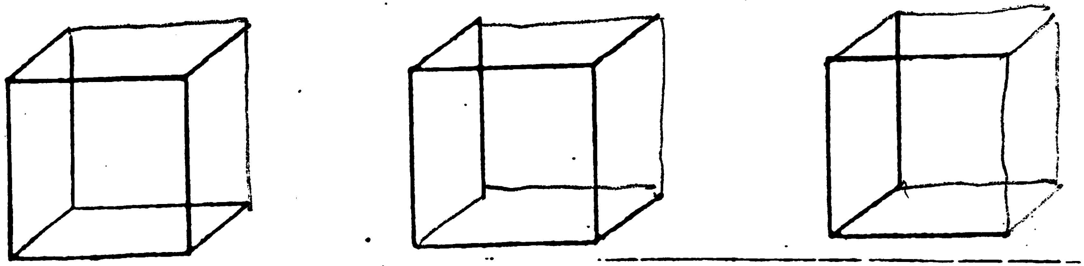

B

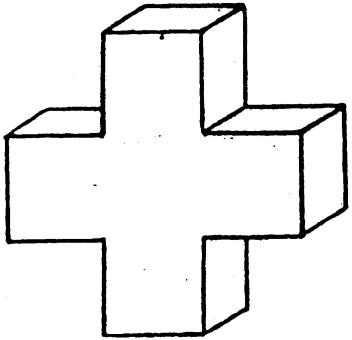

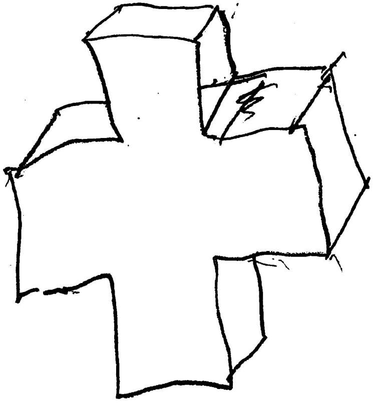

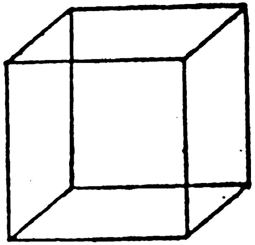

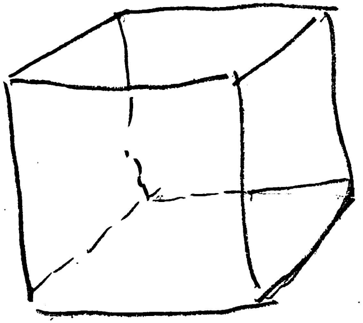

Täydennä alla olevat kuutiomallit:

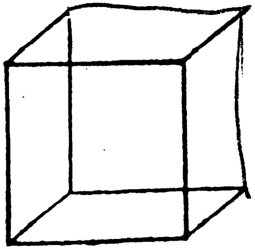

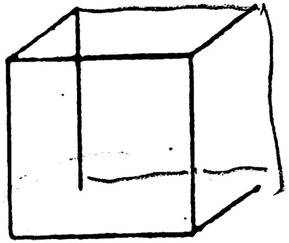

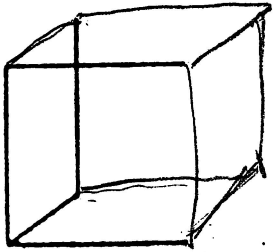

C

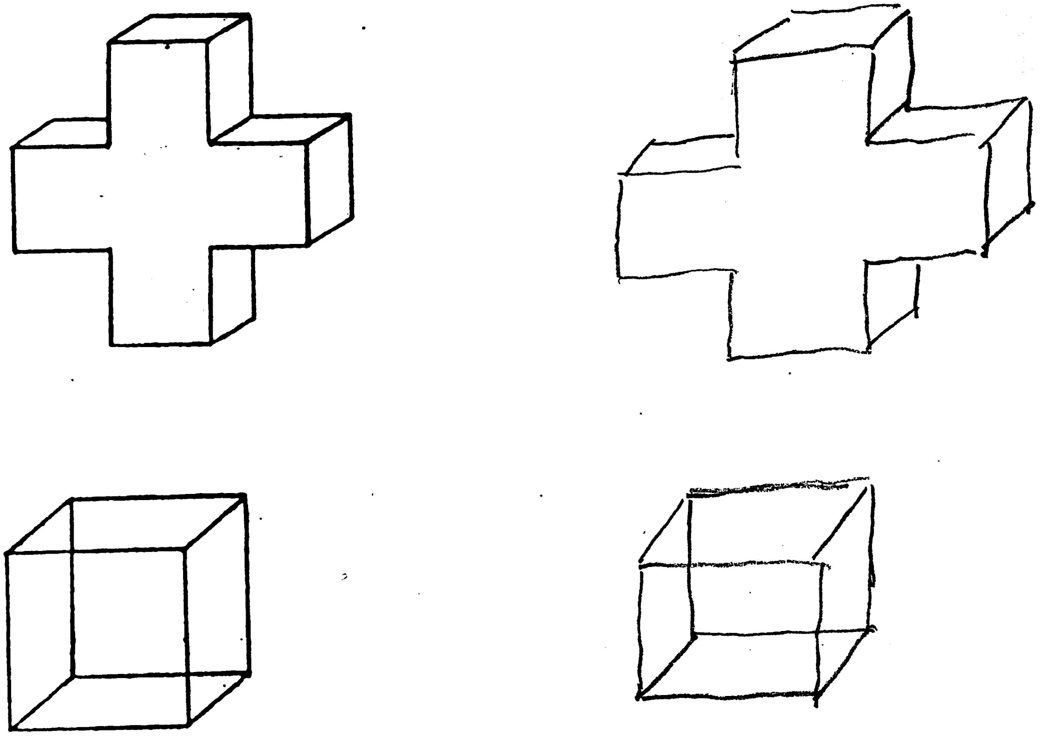

Täydennä alla olevat kuutiomallit:

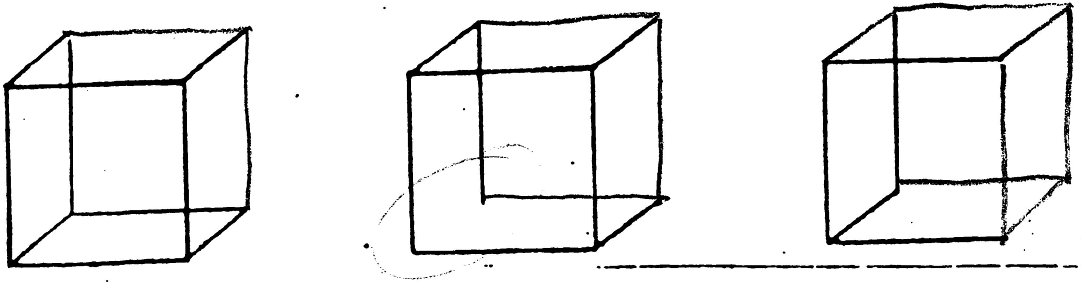

D

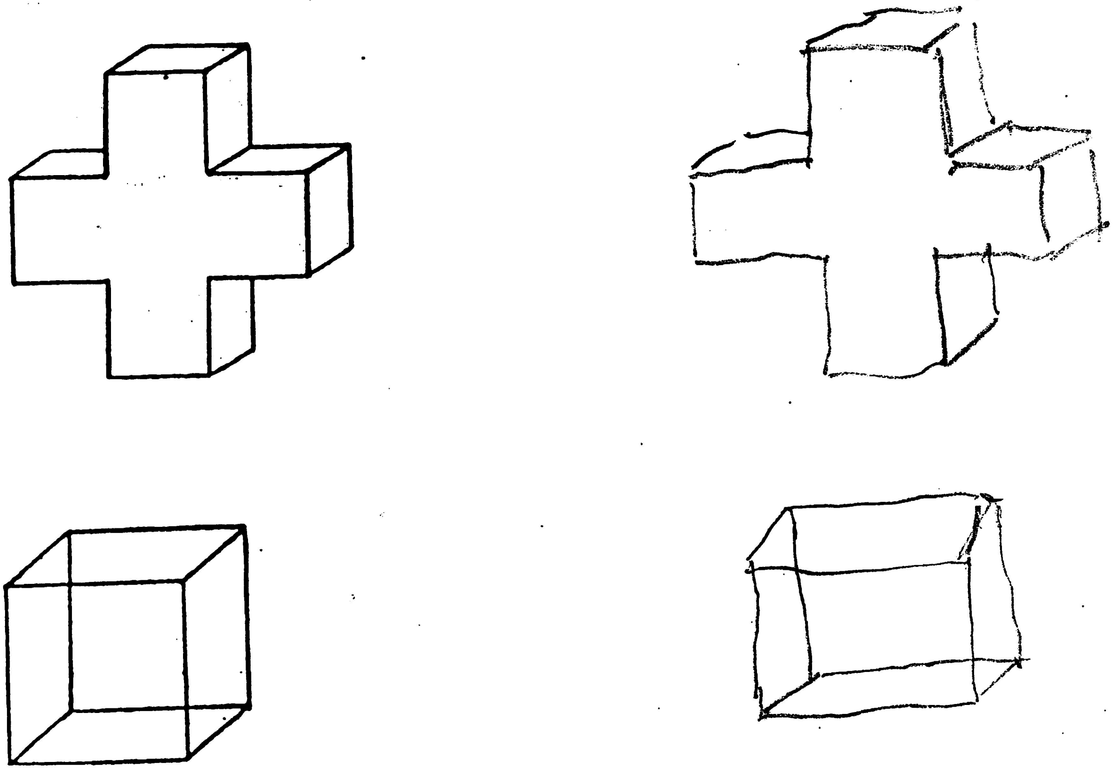

Täydennä alla olevat kuutiomallit:

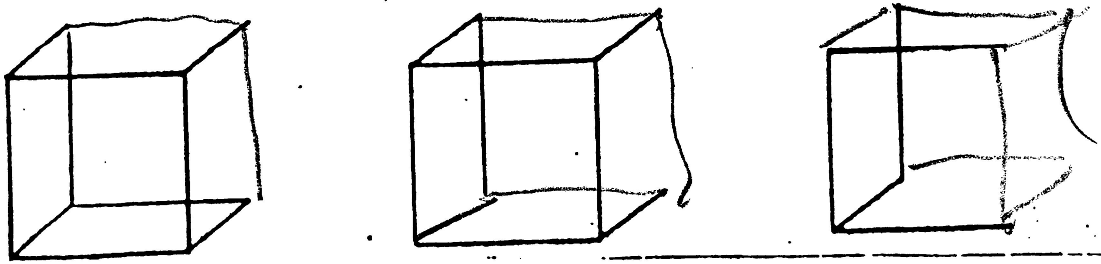

Pa

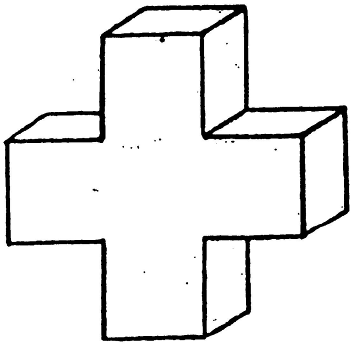

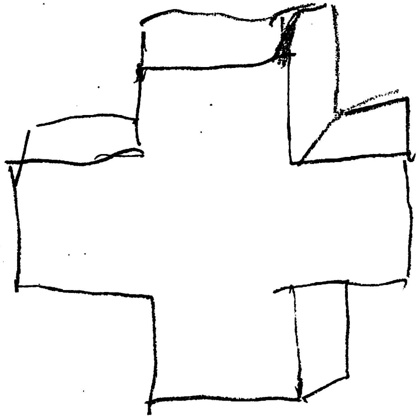

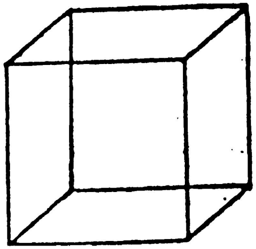

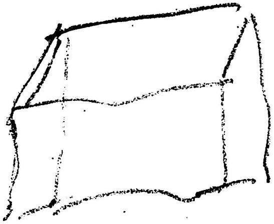

Täydennä alla olevat kuutiomallit:

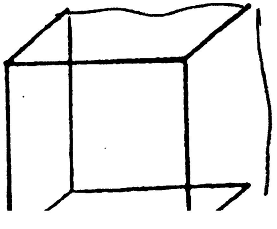

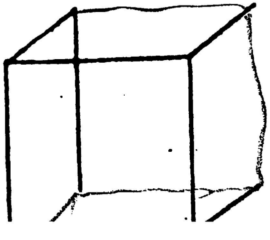

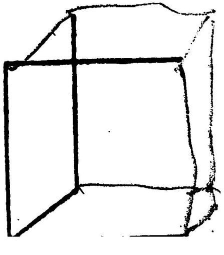

Pa

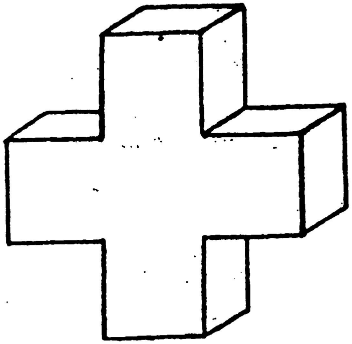

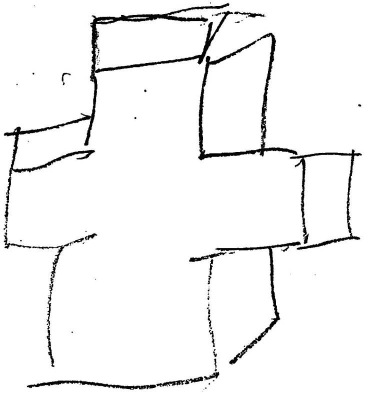

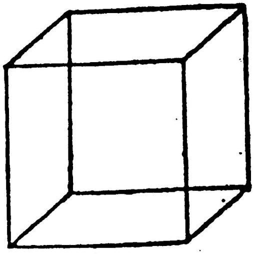

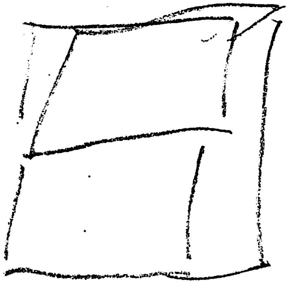

Täydennä alla olevat kuutiomallit:

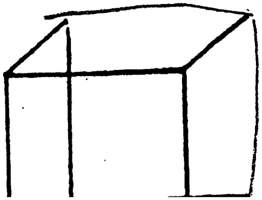

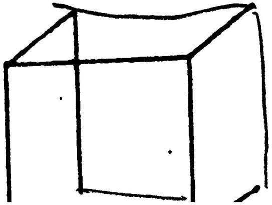

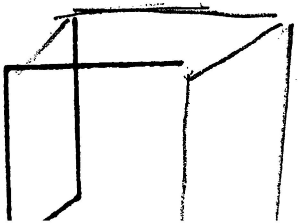

Co

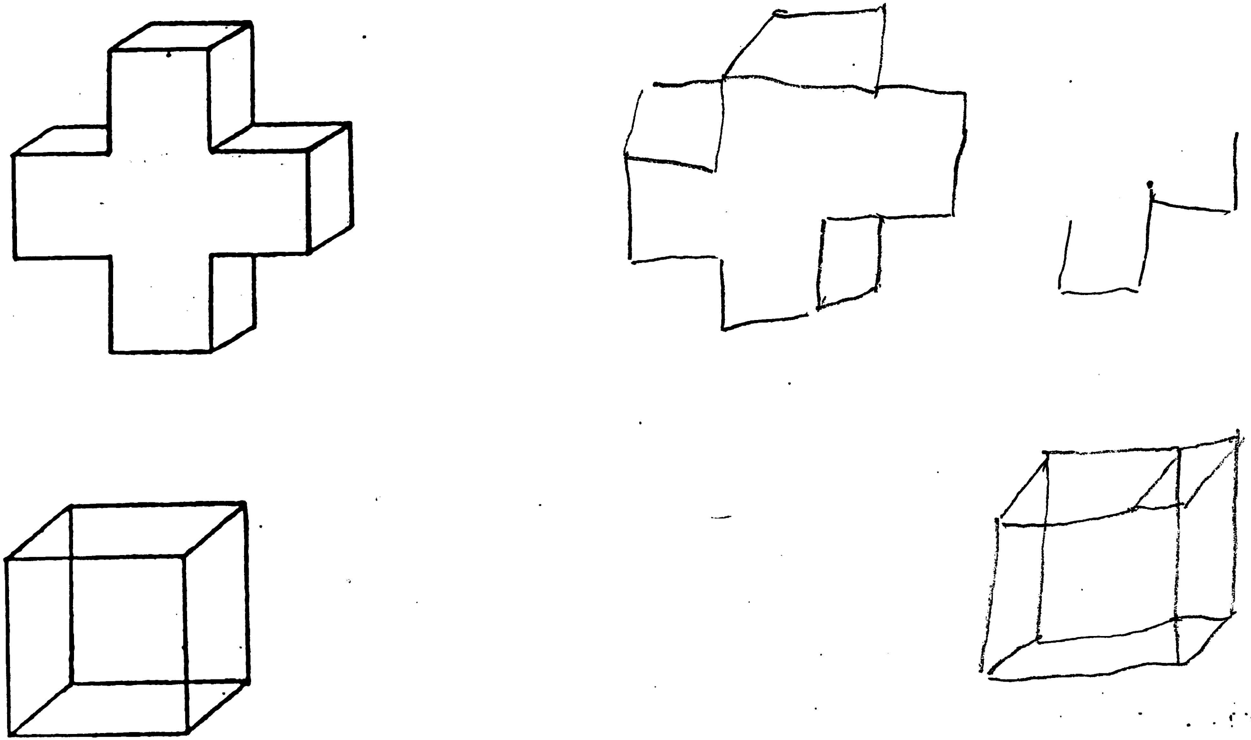

Täydennä alla olevat kuutiomallit:

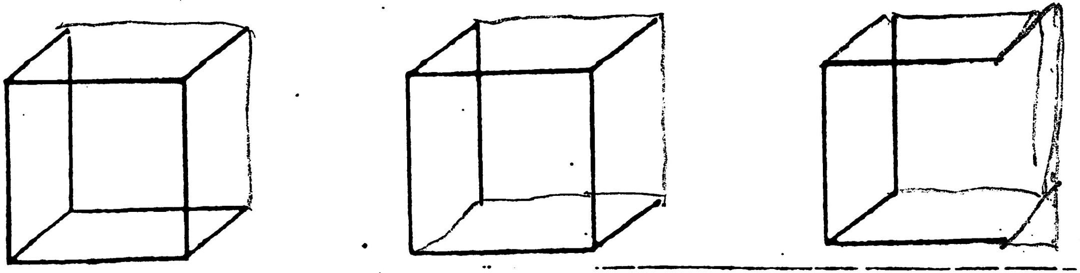

Co

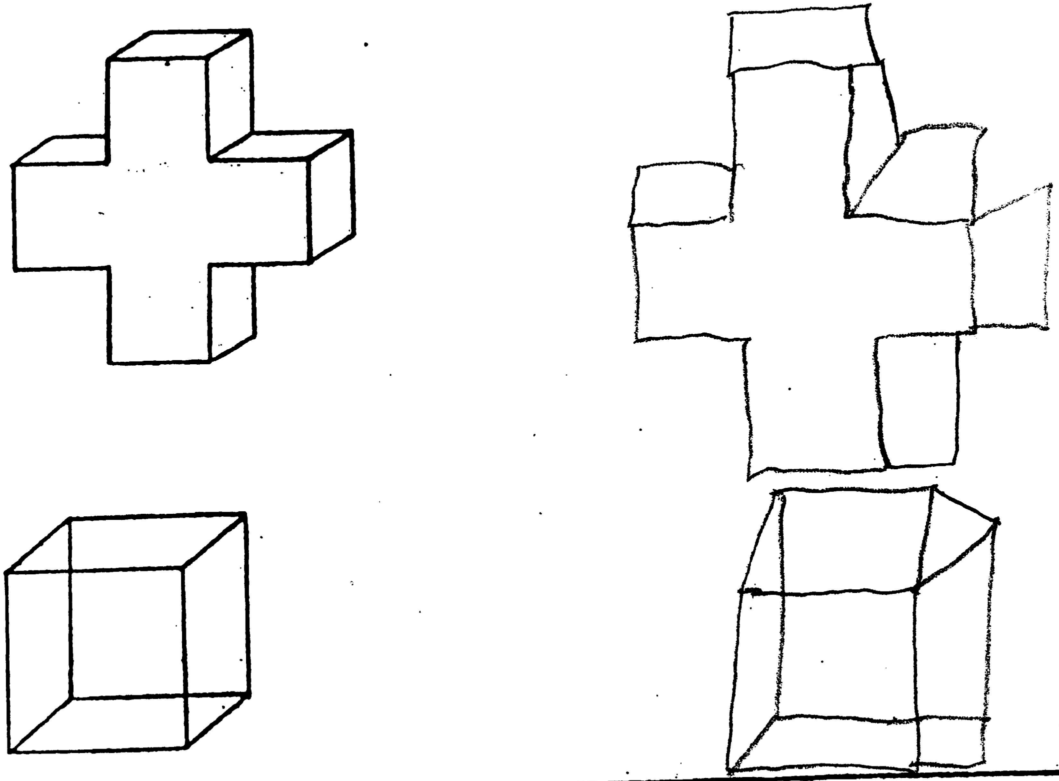

Täydennä alla olevat kuutiomallit:

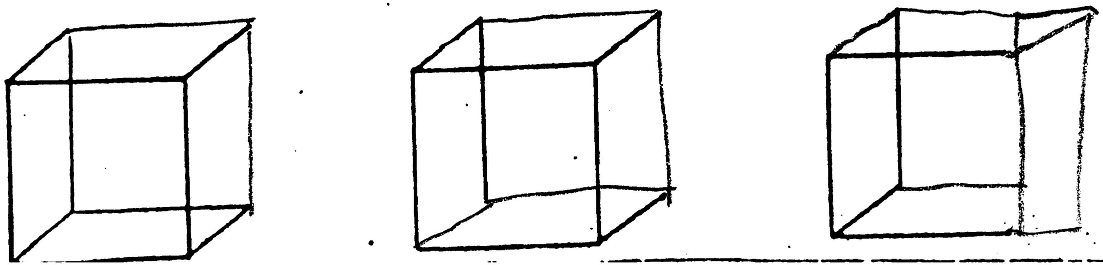

Co.

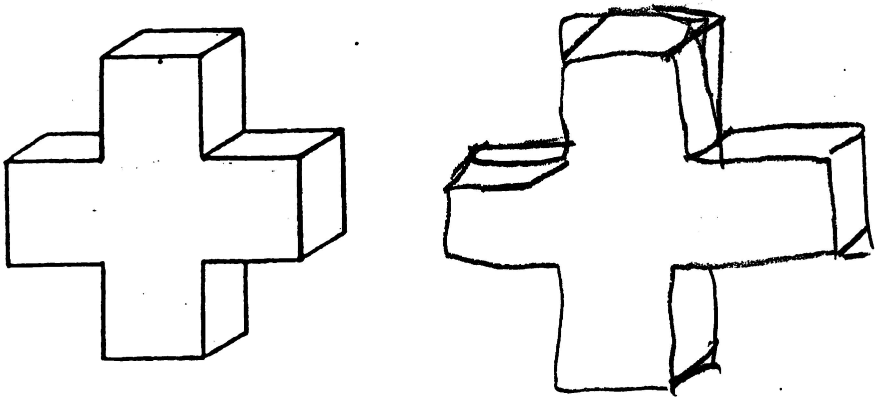

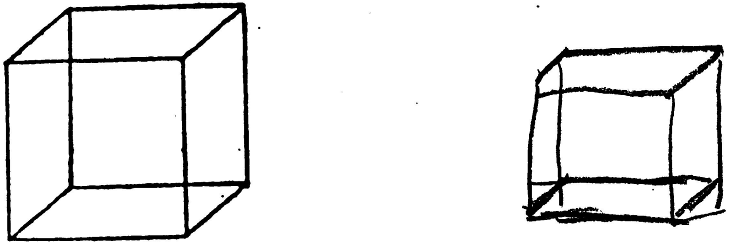

Täydennä alla olevat kuutiomallit:

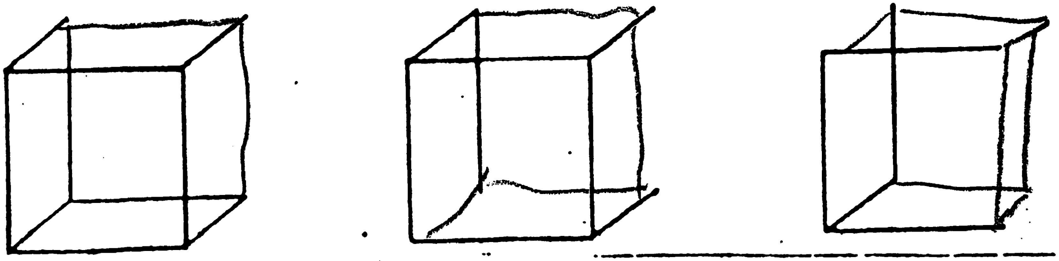

Supplement: Supplemental Information S1 — Visuoconstructional drawings of the four patients described in Table 6, (a, b, c and d, pages 1 to 4, respectively) with slight abnormality, as well as the 2 patients (pages 5 and 6) and 3 controls (pages 7 to 9) with severe abnormality. Subjects were asked to copy the Greek cross and the cube, and to fill in the missing lines of the cubes at the bottom of the page. [file peerj-02-493-s001.pdf]
